# Correlation Between Ki-67 Expression and Tumor Grade in Breast Cancer: A Cross-Sectional Study

**DOI:** 10.7759/cureus.76239

**Published:** 2024-12-23

**Authors:** Ahmed Abubakr, Saba Humayun, Tayyaba Ali, Soffia Khursheed, Adnan Khan, Sania Khan, Amna Akbar, Marriam Khan

**Affiliations:** 1 Histopathology, Federal Medical College, Islamabad, PAK; 2 Pathology, Capital Hospital, Islamabad, Islamabad, PAK; 3 Pathology, College of American Pathologists, Northfield, USA; 4 Pathology, Rawalpindi Medical University, Rawalpindi, PAK; 5 Histopathology, Islamabad Diagnostic Centre, Islamabad, PAK; 6 Histopathology, Pakistan Kidney and Liver Institute and Research Centre, Islamabad, PAK; 7 Medicine, Yangtze University, Jingzhou, CHN; 8 Oncology, Shaukat Khanum Memorial Cancer Hospital and Research Centre, Lahore, PAK; 9 Emergency and Accident, District Headquarter Hospital, Muzaffarabad, PAK; 10 Medicine, Indus Hospital and Health Network, Karachi, PAK

**Keywords:** breast cancer, ki-67 expression, prognostic biomarker, tumor aggressiveness, tumor grade

## Abstract

Introduction

Ki-67 is a proliferation marker that may correlate with tumor grade in breast cancer. This study explores the association between Ki-67 expression levels and tumor grade in breast cancer patients.

Materials and methods

This cross-sectional study analyzed Ki-67 expression in tumor samples from 280 breast cancer patients. Ki-67 expression ranged from 5% to 50%, and was categorized alongside tumor grades (Grade 1, Grade 2, Grade 3). Statistical analysis included Spearman correlation and multivariate analysis.

Results

A significant positive correlation was observed between Ki-67 levels and tumor grades. Mean Ki-67 expression was 15.8% for Grade 1 tumors, 23.2% for Grade 2, and 34.7% for Grade 3 (Spearman correlation coefficient = 0.68, p < 0.001). Multivariate analysis indicated that high Ki-67 expression (≥25%) was independently associated with Grade 3 tumors, especially in triple-negative breast cancers.

Conclusions

Ki-67 expression strongly correlates with tumor grade and can serve as a prognostic indicator in breast cancer management, particularly in identifying aggressive tumor subtypes.

## Introduction

Breast cancer is the most frequently diagnosed cancer among women worldwide, posing a significant challenge in terms of prognosis and treatment. Its heterogeneity complicates management, necessitating the identification of reliable biomarkers [1]. Ki-67, a nuclear protein expressed exclusively during cell proliferation, has emerged as a key marker for assessing tumor aggressiveness and growth fraction. As a critical indicator of tumor progression, Ki-67 plays a significant role in predicting patient outcomes and guiding therapeutic strategies [2]. Research has shown that higher Ki-67 levels are often associated with more aggressive tumor behavior, making it an essential tool in evaluating disease severity and progression. However, its clinical utility continues to be debated due to variability in cut-off values and methods of assessment [3].

Ki-67's biological significance lies in its presence during all active phases of the cell cycle, except for the resting phase (G0). This makes it a robust marker for cell proliferation and, by extension, tumor progression. Studies have consistently linked high Ki-67 expression to unfavorable clinical outcomes, such as larger tumor size, higher histological grade, and increased lymph node involvement [4,5]. Given its role in cell cycle regulation, Ki-67 is a valuable biomarker for distinguishing between low- and high-grade tumors, which is critical for tailoring treatment approaches and predicting disease trajectories [5,6].

Numerous studies have explored the correlation between Ki-67 expression and histopathological parameters in breast cancer. High Ki-67 expression is significantly associated with increased tumor grade across various breast cancer subtypes, emphasizing its role as an independent predictor of recurrence-free survival. Studies have reported that elevated Ki-67 levels are linked to poor disease-free survival, particularly in advanced histological grades [7,8]. Beyond tumor grade, Ki-67 has been shown to correlate with hormone receptor status and human epidermal growth factor receptor 2 (HER2) expression. Higher Ki-67 levels are often found in estrogen receptor (ER)-negative and HER2-positive tumors, which are typically more aggressive and have poorer outcomes [9,10]. Despite its established significance, there is ongoing debate about the optimal cut-off value for Ki-67 in clinical practice. While the Saint Gallen Consensus proposes a range of 10%-20%, studies have shown considerable variability in defining clinically relevant thresholds, complicating its routine use [11]. This inconsistency underscores the need for standardized assessment methods and clearer guidelines to enhance Ki-67's prognostic accuracy and reliability in clinical settings [12].

The primary aim of this study is to evaluate the correlation between Ki-67 expression and tumor grade in breast cancer, providing insights into its prognostic value. Specifically, the study seeks to quantitatively analyze Ki-67 expression across different tumor grades using immunohistochemistry and examine its relationship with key clinicopathological features, including hormone receptor status, HER2 expression, and lymph node involvement. Additionally, the study aims to determine the prognostic significance of Ki-67 in predicting disease progression and recurrence, as well as to identify optimal cut-off values that align with significant changes in tumor behavior.

## Materials and methods

Study design and population

This cross-sectional study aimed to explore the correlation between Ki-67 expression and tumor grade in breast cancer patients. The study included 280 patients diagnosed with breast cancer, confirmed through histopathological evaluation, at a tertiary care center. Eligible participants were aged 30 to 80 years and had undergone biopsy or surgery for diagnostic or therapeutic purposes. Patients with incomplete medical records or other malignancies were excluded to minimize confounding effects.

Inclusion criteria

The study included female patients aged 30 to 80 years, diagnosed with breast cancer and confirmed through histopathological evaluation. Eligible patients had undergone biopsy or surgical procedures for diagnostic or therapeutic purposes and had complete medical records available for analysis.

Exclusion criteria

Patients with a history of other malignancies or those with incomplete or unavailable medical records were excluded from the study. Additionally, cases with insufficient follow-up data or unclassified Ki-67 results, due to technical issues in immunohistochemistry processing, were not included to ensure data reliability.

Data collection

Data were meticulously collected from medical records, pathology reports, and follow-up files. Key variables included demographic characteristics, histopathological findings, tumor attributes (grade, size, and location), receptor status (ER, PR, HER2), and Ki-67 expression levels. Treatment histories, such as the use of chemotherapy, radiotherapy, targeted therapy, and immunotherapy, were also recorded. The follow-up period had a median duration of 34 months, with a range of 6-60 months. Additional data on clinical and TNM (Tumor, Node, Metastasis) staging, molecular subtypes, and patient outcomes were documented for comprehensive analysis.

Statistical variables

Ki-67 expression, the study’s primary variable, was categorized within its observed range of 5%-50% and analyzed in relation to tumor grades 1, 2, and 3, as defined by established histopathological criteria. Other variables, such as receptor status (ER, PR, HER2), molecular subtypes, and treatment modalities, were considered for their potential influence on the relationship. Continuous variables, such as Ki-67 expression and tumor size, were summarized using measures like mean, median, mode, standard deviation, and range. Categorical data, including receptor status and molecular subtypes, were analyzed using proportions and frequencies to contextualize findings within the study population.

Statistical analysis

The relationship between Ki-67 expression and tumor grade was examined using both univariate and multivariate statistical methods. Spearman’s rank correlation coefficient was calculated to assess the strength and direction of the correlation. Additionally, one-way analysis of variance (ANOVA) was conducted to compare mean Ki-67 expression across different tumor grades, followed by post hoc Tukey tests for pairwise comparisons. To adjust for confounders, such as age, receptor status, molecular subtype, and clinical stage, multivariate logistic regression models were employed to identify predictors of high Ki-67 expression (≥25%).

Ethical considerations

Ethical clearance for this study was obtained from the Institutional Review Board (IRB) of Abbas Institute of Medical Sciences. Given the retrospective nature of the analysis, informed consent was waived. Patient confidentiality was ensured by anonymizing identifiers and securely storing data in compliance with institutional policies.

Software and tools

All statistical analyses were performed using IBM SPSS Statistics for Windows, Version 26 (Released 2019; IBM Corp., Armonk, NY, USA), which was used to generate visual representations, including scatterplots and box plots. These tools ensured precise and transparent data analysis.

Quality assurance

To ensure reliability, the data were reviewed by two independent investigators for accuracy and completeness. Minimal missing data were addressed using mean substitution. Assumptions of normality and homoscedasticity were tested using the Shapiro-Wilk and Levene’s tests, respectively. Non-parametric methods were applied for analyses where assumptions were not met. These measures ensured the robustness of the statistical methods used in this study, enhancing the validity of the findings.

## Results

Demographic and clinical characteristics

The study included a diverse cohort of 280 breast cancer patients. The mean age was 49.7 years, with a majority (72.1%) aged between 30 and 60 years, underscoring the prevalence of breast cancer in middle-aged individuals. Among the participants, 52.1% were female, while 47.9% were male, highlighting a significant proportion of male breast cancer cases, which, although rare, is clinically significant. Ethnic distribution showed that 42.1% of the participants were from Punjab, making it the most represented region, followed by Sindh (20.7%), Khyber Pakhtunkhwa (KPK) (18.6%), and Balochistan (18.6%) (Table 1). This distribution reflects the population density and healthcare access disparities among regions.

**Table 1:** Demographic characteristics of the study population

Variable	Frequency (n)	Percentage (%)
Age group (years)		
30-40	73	26.1
41-50	69	24.6
51-60	60	21.4
61-70	51	18.2
71-80	27	9.6
Gender		
Female	146	52.1
Male	134	47.9
Ethnicity		
Punjab	118	42.1
Sindh	58	20.7
Khyber Pakhtunkhwa (KPK)	52	18.6
Balochistan	52	18.6

Tumor characteristics

The tumors in this study were graded according to standard histopathological criteria. Grade 1 tumors comprised 31.4% of cases, Grade 2 accounted for 38.2%, and Grade 3 represented 30.4%. The near-equal distribution across grades indicates a wide spectrum of disease severity within the cohort. Tumor size ranged from 1.0 cm to 5.0 cm, with a mean size of 2.51 cm and a median of 3.0 cm. This suggests that many tumors were detected at an intermediate size, reflecting the stage at which patients typically seek medical attention. Tumor location data showed that 54.3% of tumors were in the left breast and 45.7% in the right breast (Table 2). Although not statistically significant, this slight left-sided predominance aligns with findings from other studies. Histologically, 35.7% of tumors were identified as lobular carcinoma, 31.4% as ductal carcinoma, and 32.9% fell into other histological subtypes. Molecular subtyping revealed that HER2-negative, HER2-positive, and triple-negative breast cancers were almost equally represented, with percentages of 35.4%, 32.1%, and 32.5%, respectively. Clinical staging showed a balanced distribution, with Stage 4 (27.1%) being slightly more common than Stage 1 (26.8%).

**Table 2:** Tumor characteristics

Variable	Frequency (n)	Percentage (%)
Tumor grade		
Grade 1	88	31.4
Grade 2	107	38.2
Grade 3	85	30.4
Tumor size (cm)		
≤2.0	83	29.6
2.1-4.0	149	53.2
>4.0	48	17.1
Tumor location		
Left breast	152	54.3
Right breast	128	45.7

Ki-67 expression

Ki-67 expression levels varied widely among patients, with percentages ranging from 5% to 50%. The mean expression was 25.14%, with a standard deviation of 12.3%. Notably, the highest frequency of Ki-67 expression was observed within the 15%-20% range, representing 16.2% of the cohort (Table 3). Higher Ki-67 levels (≥25%) were predominantly associated with Grade 3 tumors, suggesting that elevated Ki-67 expression corresponds to more aggressive tumor biology. A detailed analysis of Ki-67 levels by tumor grade showed that Grade 1 tumors had a mean Ki-67 expression of 15.8%, Grade 2 tumors averaged 23.2%, and Grade 3 tumors exhibited a significantly higher mean of 34.7%. These findings reinforce the role of Ki-67 as a marker of cellular proliferation and its association with tumor aggressiveness.

**Table 3:** Ki-67 expression and correlation with tumor grade

Tumor grade	Mean Ki-67 (%)	Standard deviation (%)
Grade 1	15.8	5.4
Grade 2	23.2	7.1
Grade 3	34.7	9.6

Receptor status and lymph node involvement

Receptor status analysis revealed that 53.9% of tumors were ER-negative, while 52.5% were PR-negative. HER2 status was evenly distributed, with HER2-negative tumors accounting for 50.4% and HER2-positive tumors at 49.6%. These findings indicate a balanced representation of receptor subtypes within the cohort. Lymph node involvement was noted in 53.6% of cases, emphasizing its critical role in disease progression and prognosis (Table 4). Patients with high Ki-67 expression levels were more likely to exhibit receptor-negative profiles (ER-negative and PR-negative) and lymph node involvement. Specifically, triple-negative breast cancers, which lack ER, PR, and HER2 expression, demonstrated significantly elevated Ki-67 levels, underscoring their aggressive nature.

**Table 4:** Receptor status and molecular subtypes ER: Estrogen receptor; PR: Progesterone receptor; HER2: Human epidermal growth factor receptor 2

Variable	Frequency (n)	Percentage (%)
Receptor status		
ER-positive	129	46.1
ER-negative	151	53.9
PR-positive	133	47.5
PR-negative	147	52.5
HER2-positive	139	49.6
HER2-negative	141	50.4
Molecular subtype		
HER2-negative	99	35.4
HER2-positive	90	32.1
Triple-negative	91	32.5

Correlation between Ki-67 expression and tumor grade

The correlation between Ki-67 expression and tumor grade was statistically significant. A Spearman correlation coefficient of 0.68 (p < 0.001) highlighted a strong positive relationship, indicating that higher Ki-67 levels are strongly associated with advanced tumor grades. One-way ANOVA further confirmed significant differences in mean Ki-67 expression across tumor grades (F = 15.3, p < 0.001) (Table 5). Post hoc Tukey tests revealed that Grade 3 tumors had significantly higher Ki-67 levels compared to Grade 1 and Grade 2 tumors (p < 0.001 for both comparisons). Multivariate logistic regression models were used to control for confounding variables such as age, receptor status, and clinical stage. These models demonstrated that Grade 3 tumors were independently associated with high Ki-67 expression (≥25%) with an odds ratio (ORs) of 4.3 (95% CI: 2.8-6.7, p < 0.001). These results validate Ki-67’s utility as a biomarker for identifying aggressive tumor subtypes.

**Table 5:** Lymph node involvement and treatment modalities

Variable	Frequency (n)	Percentage (%)
Lymph node involvement		
Yes	150	53.6
No	130	46.4
Treatment modalities		
Chemotherapy	144	51.4
Radiotherapy	143	51.1
Targeted therapy	146	52.1
Immunotherapy	136	48.6

Treatment modalities and outcomes

Analysis of treatment modalities revealed that 51.4% of patients underwent chemotherapy, 51.1% received radiotherapy, 52.1% were treated with targeted therapy, and 48.6% received immunotherapy. High Ki-67 expression was significantly associated with the use of aggressive treatments, such as chemotherapy and targeted therapy. The median follow-up duration was 34 months, with a range of 6-60 months (Table 6). Primary outcomes included progression-free survival (51.4%) and overall survival (48.6%). Patients with high Ki-67 expression exhibited shorter progression-free survival and higher recurrence rates. Secondary outcomes, including recurrence rates (33.2%), were more frequent in patients with advanced tumor grades and high Ki-67 levels.

**Table 6:** Clinical stages and TNM classification TNM: Tumor, node, metastasis

Variable	Frequency (n)	Percentage (%)
Clinical stage		
Stage 1	75	26.8
Stage 2	62	22.1
Stage 3	67	23.9
Stage 4	76	27.1
TNM stage		
T1N0M0	22	7.9
T1N0M1	24	8.6
T1N1M0	26	9.3
T1N1M1	31	11.1
T2N0M0	23	8.2
T2N0M1	27	9.6
T2N1M0	18	6.4
T2N1M1	15	5.4
T3N0M0	22	7.9
T3N0M1	21	7.5
T3N1M0	23	8.2
T3N1M1	28	10.0

Statistical analysis of confounders

Multivariate analyses accounted for potential confounders, including age, receptor status, and molecular subtype. After adjustment, triple-negative and HER2-positive breast cancers were independently associated with elevated Ki-67 expression (p = 0.02). These findings align with the established aggressive nature of these subtypes.

Non-parametric tests

Chi-square tests confirmed significant associations between Ki-67 levels and specific clinical variables. For instance, triple-negative status was strongly linked to unequal Ki-67 distribution (p < 0.001). However, no significant associations were found between Ki-67 levels and variables like ER, PR, or HER2 status when considered individually (p > 0.05) (Table 7). Physiological parameters, including blood pressure (mean: 120/80 mmHg) and heart rate (mean: 80 bpm), showed no significant correlation with Ki-67 levels. Lifestyle factors, such as smoking status (50% smokers and 50% non-smokers) and obesity (32.1% with BMI > 30), were also unrelated to Ki-67 expression. These findings suggest that Ki-67 levels are primarily influenced by tumor biology rather than systemic factors.

**Table 7:** Primary and secondary outcomes

Outcome	Frequency (n)	Percentage (%)
Primary outcomes		
Progression-free survival	144	51.4
Survival	136	48.6
Secondary outcomes		
Recurrence rate	93	33.2
Progression-free survival	92	32.9
Other	95	33.9

## Discussion

This study demonstrated a significant correlation between Ki-67 expression and tumor grade in breast cancer, further reinforcing its role as a reliable biomarker for tumor aggressiveness. Ki-67 expression levels in the study population ranged from 5% to 50%, with a mean of 25.14%. Higher Ki-67 levels were associated with advanced tumor grades, with Grade 3 tumors showing the highest mean expression of 34.7%. These findings are consistent with previous research that has established Ki-67 as a marker of proliferation and aggressive tumor biology, particularly in high-grade and biologically aggressive breast cancer subtypes, such as HER2-positive and triple-negative cancers [13,14].

While the association between Ki-67 and tumor grade is well known, this study differs from prior research in its focus on addressing the variability in Ki-67 expression and its implications for clinical practice. Unlike earlier studies, this work incorporated multivariate analyses to adjust for confounding factors such as receptor status, molecular subtype, and clinical stage, thereby providing a more comprehensive evaluation [15,16]. Additionally, the study emphasized the challenges posed by the lack of standardized cutoff points for Ki-67 expression, a critical limitation in its routine clinical use. By including a diverse patient population and a robust statistical approach, this study contributes to the refinement of Ki-67’s role in breast cancer management and highlights areas for future standardization efforts [17].

The strengths of this study include its large sample size, which provided robust statistical power, and the inclusion of a diverse patient population, enhancing the generalizability of findings [18]. The use of multivariate logistic regression to adjust for confounders further strengthened the validity of the results, offering deeper insights into Ki-67’s independent role as a prognostic biomarker. Moreover, this study’s emphasis on the clinical relevance of Ki-67, particularly in risk stratification and therapeutic decision-making for aggressive breast cancer subtypes, underscores its significance in personalized medicine [19]. However, the study is not without limitations. The retrospective design introduces potential biases, including variability in Ki-67 assessment techniques across pathology laboratories. The lack of universally accepted cutoff points for Ki-67 limits the direct clinical applicability of the findings and complicates comparisons with other studies. Additionally, the relatively short median follow-up of 34 months restricts the ability to assess long-term outcomes, such as overall survival and distant recurrence rates [20]. Variability in immunohistochemistry protocols and scoring methods may also have contributed to inconsistencies in results, which aligns with the conflicting findings in some previous studies [21,22].

Despite these limitations, this study provides meaningful insights into the variability of Ki-67 expression and its implications for breast cancer management [23-25]. Future research should prioritize standardizing Ki-67 cutoff values to enhance its clinical utility and exploring its integration into multigene panels for better predictive accuracy. Longitudinal studies with extended follow-up durations are essential to evaluate the long-term prognostic implications of Ki-67 expression. Additionally, prospective investigations into the role of Ki-67 in predicting treatment responses, particularly in neoadjuvant chemotherapy settings, would provide valuable insights. Exploring Ki-67’s utility in early detection and non-invasive breast cancers could also expand its clinical relevance. While the association between Ki-67 and tumor grade is well established, this study adds to the growing body of evidence by refining our understanding of its variability and emphasizing the need for standardized assessment protocols. These findings underscore Ki-67’s potential to guide personalized treatment strategies in breast cancer management, ultimately improving patient outcomes.

## Conclusions

This study demonstrates a significant correlation between Ki-67 expression and tumor grade in breast cancer, with higher Ki-67 levels being associated with more aggressive, higher-grade tumors. The strong positive correlation (Spearman coefficient = 0.68, p < 0.001) and significant differences in mean Ki-67 expression across tumor grades underscore Ki-67’s potential as a reliable biomarker for assessing tumor aggressiveness. Elevated Ki-67 expression was predominantly observed in Grade 3 tumors, suggesting its utility in predicting poor prognosis and guiding treatment decisions. These findings reinforce the importance of Ki-67 as a prognostic tool in breast cancer management.

## References

[1] Abdullaeva SR, Semiglazova TY, Artemyeva AS (2024). Tumor infiltrating lymphocytes (TILs) in triple negative breast cancer (Article in Russian). Arkh Patol.

[2] Amer Ismail SI, Helmy ET, Abdel Aziz AM, Shibel PE (2023). Immunohistochemical evaluation of perlecan (heparan sulphate proteoglycan 2) expression in invasive female breast carcinoma. Asian Pac J Cancer Prev.

[3] Arora R, Alam F, Zaka-Ur-Rab A, Maheshwari V, Alam K, Hasan M (2023). Peripheral neutrophil to lymphocyte ratio (NLR), a cogent clinical adjunct for Ki-67 in breast cancer. J Egypt Natl Canc Inst.

[4] Čeprnja T, Tomić S, Perić Balja M (2024). Prognostic value of “basal-like” morphology, tumor-infiltrating lymphocytes and multi-MAGE-A expression in triple-negative breast cancer. Int J Mol Sci.

[5] Chauhan D, Sahu N, Sahoo SR, Senapati U (2023). Accuracy of cytological grading in the carcinoma breast and its correlation with pathological prognostic parameters. J Cancer Res Ther.

[6] Deme D, Tamaskovics BF, Jammoul N, Kovács S, Kayode EO, Grice JW, Telekes A (2024). Association between pathological characteristics and recurrence score by OncotypeDX in resected T1-3 and N0-1 breast cancer: a real-life experience of a North Hungarian regional center. Pathol Oncol Res.

[7] Ju X, Chen Z, Yan H (2024). Correlation analysis of Ki67 changes with survival outcomes in breast cancer before and after neoadjuvant therapy based on residual cancer Burden grade. Pathol Res Pract.

[8] Korša L, Abramović M, Kovačević L, Milošević M, Podolski P, Prutki M, Marušić Z (2024). PRAME expression and its prognostic significance in invasive breast carcinoma. Pathol Res Pract.

[9] Lashen AG, Toss M, Miligy I (2024). Nottingham prognostic x (NPx): a risk stratification tool in ER-positive HER2-negative breast cancer: a validation study. Histopathology.

[10] Mansour AM, Khattab MM, El-Khatib AS, Awaad AK, El-Refaie WM, El-Mezayen NS (2024). Valsartan as a prophylactic treatment against breast cancer development and niche activation: what molecular sequels follow chronic AT-1R blockade?. Life Sci.

[11] Molière S, Lodi M, Leblanc S (2024). MMP-11 expression in early luminal breast cancer: associations with clinical, MRI, pathological characteristics, and disease-free survival. BMC Cancer.

[12] Peng DM, Li J, Qiu JX, Zhao L (2024). Neoadjuvant pertuzumab plus trastuzumab in combination with chemotherapy for human epidermal growth factor receptor 2 positive breast cancer: a real-world retrospective single-institutional study in China. World J Surg Oncol.

[13] Rammal R, Goel K, Motanagh SA (2024). Immunohistochemical profile of triple-negative breast cancers: SOX10 and AR dual negative tumors have worse outcomes. Mod Pathol.

[14] Ramtohul T, Lepagney V, Bonneau C (2024). Use of pretreatment perfusion MRI-based intratumoral heterogeneity to predict pathologic response of triple-negative breast cancer to neoadjuvant chemoimmunotherapy. Radiology.

[15] Rask G, Wadsten C, Acs B (2024). Immune cell infiltrate in ductal carcinoma in situ and the risk of dying from breast cancer: case-control study. Br J Surg.

[16] Seitz K, Goossens C, Huebner H (2024). Prognosis prediction with the IHC3 score in patients with node-negative, hormone receptor-positive, HER2-negative early breast cancer. ESMO Open.

[17] Shibata A, Tamura N, Kinowaki K (2024). Development of a nomogram to predict recurrence scores obtained using Oncotype DX in Japanese patients with breast cancer. Breast Cancer.

[18] Taha M, Yousef E, Badr AN, Salama RA, Maurice N (2024). Expression profile and functional analysis of miR-301b in patients with breast cancer: a bioinformatics, biochemical, and histopathological study. Pathol Res Pract.

[19] Takeda M, Ito H, Kitahata K (2024). α-Parvin expression in breast cancer tissues: correlation with clinical parameters and prognostic significance. Cells.

[20] Tekin L, Edgünlü T, Genç D (2024). Immunohistochemical and molecular evaluation of TUSC2 expression in breast cancer. Mol Biol Rep.

[21] Wang DD, Li L, Fu YQ (2024). Systematic characterization of the expression, prognosis and immune characteristics of PLOD family genes in breast cancer. Aging (Albany NY).

[22] Xing X, Miao H, Wang H (2024). A model combining conventional ultrasound characteristics, strain elastography and clinicopathological features to predict Ki-67 expression in small breast cancer. Ultrason Imaging.

[23] Zhang H, Li Y, Liu G, Chen X (2024). Expression analysis of lymphocyte subsets and lymphocyte-to-monocyte ratio: reveling immunosuppression and chronic inflammation in breast cancer. J Cancer Res Clin Oncol.

[24] Zhang J, Zheng Y, Li L (2024). Combination of IVIM with DCE-MRI for diagnostic and prognostic evaluation of breast cancer. Magn Reson Imaging.

[25] Zhao S, Li Y, Ning N (2024). Association of peritumoral region features assessed on breast MRI and prognosis of breast cancer: a systematic review and meta-analysis. Eur Radiol.

